# Identifying the systemically important banks of Turkey with the CoVaR method

**DOI:** 10.1016/j.heliyon.2020.e04790

**Published:** 2020-09-01

**Authors:** Zehra Civan, Gulhayat Golbasi Simsek, Ebru Caglayan Akay

**Affiliations:** a Vakifbank, Saray Mah. Dr. Adnan Buyukdeniz Cad. No:7/A-B Umraniye, 34768, Istanbul, Turkey; b Department of Statistics, Faculty of Arts & Science, Yildiz Technical University, 34220, Istanbul, Turkey; c Department of Econometrics, Faculty of Economics, Marmara University, 34722, Istanbul, Turkey

**Keywords:** Systemic risk, Systemically important bank, Conditional value-at-risk, Quantile regression, Statistics, Finance, Banking, Macroeconomics, Microeconomics, Econometrics

## Abstract

The purpose of this paper is to measure the systemic risk contributions of Turkish banks and to identify the systemically important banks of Turkey during the period from 2005 to 2016. We apply the conditional value-at-risk (CoVaR) method proposed by Adrian and Brunnermeier (2009) using quantile regression. The study includes thirteen major banks of Turkey, including both public and private banks, out of a total of 52 banks. The banks are ranked in terms of their systemic risk contribution to the Turkish financial system based on their asset returns, macroeconomic variables and individual bank variables. The study reveals that Akbank, Garanti, Yapi Kredi and Isbank have the highest systemic risk contribution to the financial system when adding macroeconomic variables to the model. This ranking is changed to Yapi Kredi, Garanti, TEB, Sekerbank and Akbank when taking into account bank-specific variables. One surprising result is that risk in isolation and the spillover risks of public banks are smaller than in large private banks. Furthermore, the marginal systemic risk contributions of public banks are smaller than those of private banks. In conclusion, authorities improve the regulatory framework according to the context of CoVaR in addition to monitor the idiosyncratic risks of banks.

## 1 Introduction

With the last financial crisis starting at the end of 2007 and labelled as a crisis in 2008, measuring and managing systemic risk and the methods used for this evaluation have become important subjects in academia and for the institutions and authorities regulating the financial sector. When institutions that cause systemic risk are considered, banks are typically the focus. Accordingly, the general opinion is that the banks are the financial institutions that pose almost the entire systemic risk. When, how and why banks pose a systemic risk is examined, and a general opinion regarding financial markets is reached.

There is no single determinant for the recent crisis; rather, it was caused by many factors acting in concert. Accordingly, applying only one precaution will not be sufficient to prevent systemic risk. Thus, it is necessary to take many precautions at the same time and consider their effects on each other. When the conducted studies are reviewed, the size of the financial institution is considered one of the important determinants of systemic risk. In addition to size, the domino effect along with interconnectedness and contagiousness are common determinants mentioned in almost all related studies.

Especially in conjunction with the 2008 crisis, the issue of too-big-to-fail institutions constituted a big problem for countries. Bernanke (2009) stated that when large financial institutions that are interconnected with other institutions are unsuccessful and face issues during crisis periods, relevant authorities make sacrifices and provide incentives. As the distress of such institutions will contain risks for the financial system and the real economy in a broad sense, the relevant authorities do not allow bankruptcies of such institutions (Ennis and Malek, 2005). However, institutions that are too big to fail have many undesirable effects; for example, they adversely affect market discipline, increase the capital shortfall of the market by borrowing more loans with less capital, and distort competition with smaller institutions. Moreover, rating institutions may give high scores to such institutions which obtain public guarantees. Indeed, according to Moody's, fifty major banks were supported in 2009, which increased their ratings three grades in that year (BIS, 2010).

When a bank faces trouble in the financial network among banks, it creates a domino effect and affects other financial institutions negatively to a certain (smaller or larger) degree. For example, the liquidity risk arising due to the problem of trust between banks creates a domino effect and may generate systemic risk by spreading to other banks. With the recent financial crisis, this situation of interconnectedness or mutual interdependence is one of the determinants generating the systemic risk. There exists a growing literature that examines the effect of interconnectedness in the financial system. Billio et al. (2012) propose several econometric measures of systemic risk to capture interconnectedness among the largest institutions in the finance and insurance sectors. Segoviano and Goodhart (2009) focus on the financial linkages through distress dependencies among the banks in the system using credit default swaps data, see also Roengpitya and Rungcharoenkitkul (2011) who examine the systemic risk contribution and financial linkages through a sample of Thai banks using CoVaR.

Another determinant, i.e., the contagion effect, is inherent in systemic risk and begins when systemic risk occurs (Martinez-Jaramillo et al., 2010). Smaga (2014) defined the contagion effect as the possibility that a financial institution, instrument, market, substructure or financial-system-based instability will spread to the other parts of the financial system and create a crisis affecting the whole system.

In the literature focusing on the precautions to reduce systemic risk, many recommendations have been offered; Bernanke (2009) summarized these suggestions under four main categories in his speech at the Financial Stability Forum on March 10, 2009: too big to fail, empowerment of the financial substructure, cyclicality of the supervision system and establishment of a systemic risk authority.

A financial crisis that arises from large-scale financial institutions that are too big to fail will spread to other economies at the global level and have a negative effect. In the literature, it is possible to find several kinds of studies on examining the relationship between large financial institutions and systemic risk. For example, Borri et al. (2012) investigate the systemic risk contributions of 223 European listed banks. They find that bank size and leverage are two predictors of the systemic risk contributions of banks. Lahmann and Kaserer (2011) examine the systemic risk of 83 international banks, and the results confirm the common statement that the banks defined as too-big-to-fail impose larger systemic risk. Similar results are found in the studies by Huang et al. (2011), Adrian and Brunnermeier (2009) and Roengpitya and Rungcharoenkitkul (2011). To prevent this outcome, the relevant authorities should prevent systemically important financial institutions from taking excessive risks (Bernanke, 2009). Certain actions are needed to ensure the effective supervision of big institutions with complex natures. First, weak institutions in terms of capital sufficiency, liquidity and risk management should be detected by the supervisory authorities. Large institutions should be monitored and supervised regularly. Any issues resulting from companies going bankrupt should be resolved, and their cost to the economy should be minimized. An institution providing a greater systemic risk over others should be improved in terms of its capital sufficiency and liquidity rates. The financial substructure must be further improved.

A review of the existing literature reveals that systemic risk has been extensively researched in the markets of European countries and many other developed markets, whereas the research on systemic risk related to the Turkish financial sector is highly limited. In general, the existing literature deals with measuring the systemic risk of the Turkish banking sector based on only the stock returns and examining the determinants of systemic risk of the Turkish financial market, not on a bank level, see details in Section 2. We could not find a complete study that has examined the impact of individual bank characteristics of Turkish banks and macroeconomic variables on the systemic risk contribution of individual Turkish banks. Thus, the present study was conducted to fill these research gaps.

To identify the systemically important banks of Turkey, the CoVaR method proposed by Adrian and Brunnermeier (2009) was applied in this study. The contribution of the study is to present a solid investigation of the Turkish banking sector, taking into account macroeconomic variables and individual bank variables in addition to the asset returns of banks in order to measure the marginal systemic risk contribution of Turkish banks. Then, all the results are compared between public banks and private banks, and the banks are ranked with respect to their systemic risk contribution to the system. The largest bank of Turkey, Ziraat, is included in this study for the first time in order to measure its marginal systemic risk contribution to the financial system based on macroeconomic variables and its bank variables demonstrating its balance sheet characteristics.

The remainder of the paper is structured as follows: Section 2 presents a literature review and some general information about the Turkish banking sector. Section 3 describes the dataset. Section 4 discusses the CoVaR methodology and the contribution to systemic risk (denoted by ΔCoVaR). Section 5 presents the empirical results. In Section 6, the conclusions and a discussion are given.

## 2 Literature review

Systemic risk, in its simplest sense, can be defined as the potential collapse of a part or the entire financial system. However, there is no single definition of systemic risk that can be agreed upon. Instead, several alternative definitions have been proposed in the literature.

A well-known definition is presented by Mishkin (1995) who defines systemic risk as the odds of an unexpected event that negatively affects the parties of the most efficient investment opportunities regarding their access to funding channels and interrupts the financial markets. Kaufman (1995) defines the risk of distress that could be suffered by financial institutions interconnected with the domino effect as a result of a systemic risk chain reaction. Bartholomew and Whalen (1995) describe systemic risk as a situation that affects the entire banking system, financial system or the economic system rather than one or a few institutions. De Bandt and Hartmann (2000) explain it as the result of a systemic crisis that causes the collapse of the entire financial system by negatively and substantially affecting a significant proportion of financial companies and financial markets. Erdem Basci, who was the President of the Central Bank of the Republic of Turkey (CBRT) between April 2011 and April 2016, gave the opening speech at the G-20 summit organized in Istanbul on September 27, 2012, titled “Financial Systemic Risk”, and emphasized the contagiousness of systemic risk. Basci explained the concept of systemic risk by underlining the mutual interconnectedness among financial institutions.

Although it has no academic definition, for any risk to be a systemic risk, it has to include one, more than one, or all of the following properties. Although these properties differ in reference to the market conditions of every country, they can be designated as common properties.

• Adversely affecting the majority of the financial system or the whole system,
• Interruption of all or a substantial part of financial services,
• Causing a loss of trust in financial markets,
• Adversely affecting the economy or society's welfare,
• Interruption or stopping of payment systems or loan flow, and
• Sudden and unexpected drop in asset prices.

The literature consisting of theoretical and empirical studies on systemic risk is gradually being enriched. When examining the literature, there is no one standard method used to measure systemic risk. This risk is measured with the help of different approaches. Lehar (2005) proposes the default probabilities of financial institutions based on Merton's model as a measurement method of systemic risk. Huang et al. (2009) use data on credit default swaps and equity return correlations to estimate systemic risk as the price of insurance against financial crisis. Acharya et al. (2010) suggest two new approaches, namely Systemic Expected Shortfall (SES) and Marginal Expected Shortfall (MES), to estimate the realized systemic risk contribution of individual financial institutions. Systemic expected shortfall is defined as its propensity to be undercapitalized when the entire system is undercapitalized. Marginal expected shortfall and leverage are used to calculate the systemic expected shortfall of institutions. This method was developed by Engle and Brownlees (2011) proposed Systemic Risk Index (SRISK) to measure the systemic risk contribution of an institution. SRISK measures the expected capital shortfall of an institution conditional on its size, leverage and risk. Alternatively, Tarashev and Zhu (2011) and Cao (2013) suggest a new approach to predict the systemic risk of financial institutions using Shapley values. Gray and Jobst (2013) examine contagion across markets and institutions using contingent claims analysis. Zhou (2010) uses the multivariate extreme value theory framework to provide two measures of systemic risk, the systemic impact index and the vulnerability index, which assess the risk that an institution imposes to the system and the risk that the system imposes to the institution. Billio et al. (2012) use principal component analysis and Granger-causality tests to measure connectedness and systemic risk. Zhu et al. (2019) research the relationship between information disclosure and systemic risk in the presence of a deposit insurance system as well as their multiplicative effects on systemic risk through a dynamic game. They apply an empirical test using panel data on different countries listed commercial banks. Adrian and Brunnermeier (2009) propose a new method, CoVaR, to measure spillover effects and the systemic risk contribution of individual institutions. This method is based on estimating the value-at-risk (VaR) of the financial system conditional on an institution being in distress. This method focuses on estimating the market valued of total assets of financial institutions. The CoVaR method differs from the other approaches such as the approach of Lehar (2005) that focuses on estimating the probability of default of bank's asset portfolios.

The CoVaR method has been applied in many studies and extended by alternative approaches. For example, Girardi and Ergun (2013) change the definition of financial distress as an institution being at most at its VaR level. They estimate time varying CoVaR by employing a three-step procedure using multivariate GARCH method. Cao (2013) extends the original CoVaR model to more than one institution being in financial distress. Multi-CoVaR which measures the systemic risk conditional on several institutions facing financial difficulties at the same time was used in this study. Reboredo and Ugolini (2015) apply the CoVaR method using copula approach to examine the systemic risk in European sovereign debt markets. Quyang et al. (2020) apply a directed network approach to measure systemic risk contagion effect of Chinese banking industry. Yun and Moon (2014) examine systemic risk in the Korean banking sector by applying two well-known systemic risk measures, the MES and CoVaR. To compute both measures, they use the dynamic conditional correlation models which are types of multivariate GARCH models.

A few studies have investigated the systemic risk of Turkish banks, in which the indicators-based method suggested by the Basel Committee on Banking Supervision (BCBS) was preferred. Sacci and Sayılgan (2014) rank twenty-eight Turkish banks with respect to their systemic risk by using the indicators-based method. Tunay (2015) examines Turkish banks’ systemic risk with respect to the approach of systemic risk index, called SRISK, by using linear panel data analysis. He concludes that the individual risks of the large-scale commercial banks of Turkey are affected by their capital adequacy ratios, leverage ratios and size, such as their total assets. Karadag (2015) investigates the last global financial crisis, the concept of systemic risk and especially the examples of rescued institutions in the countries affected by the last crisis. Talasli (2013) applies systemic expected shortfall to examine the systemic risk of the Turkish financial institutions using their stock returns, leverage, stock market beta and annualized volatility of stock returns. The results concluded that SES is a powerful systemic risk alternative approach. Binici et al. (2013) survey the development of systemic risk in the Turkish banking sector using co-movement of the stock returns of banks and search the possible determinants of systemic risk. As a result, they find the main determinants of systemic risk: the market share of bank pairs, the amount of non-performing loans, heard of behavior of banks, and the volatilities of macrovariables. Chadwick and Ozturk (2018) establish 15 different single financial stress indicators for Turkey using principal component analysis, basic portfolio theory, variance equal weights and Bayesian dynamic factor model. They use 14 variables representing five different markets and attempted to find a best indicator for Turkey and compare the results. Akkoyun et al. (2013) measure the systemic importance of eight Turkish banks with contingent claims analysis and Shapley value without giving a name. Sengul and Yilmaz (2019) investigate the systemic risk of the Turkish banking sector by using CoVaR and marginal expected shortfall. The data includes six Turkish banks during 2000–2016 and included macroeconomic and financial market variables. However, they examine and rank the systemic importance of these six banks in terms of only their stock loss. They do not consider the effects of macroeconomic variables and bank-specific characteristics on their individual systemic importance.

The existing literature on the systemic risk of Turkish banks are mainly on measuring the systemic risk of the financial market instead of examining on a bank level. Therefore, this study contributes the literature by investigating the systemic risk of Turkish banks on a bank level with the CoVaR method in a broad sense. In the CoVaR method proposed by Adrian and Brunnermeier (2009), a parametric regression model is used as a functional form of the model. The functional form of the models in this study are checked and determined to be linear and used the approach of Adrian and Brunnermeier (2009). When considering the nonparametric structure of financial risk and the contagion effect, it is applied to different approaches. For example, Quyang et al. (2020) use a single index model to represent the semi-parametric CoVaR model through a directed network approach. Besides, we examine the systemic risk contribution of each bank one-by-one in this paper; thus, it is different from the studies investigating the systemic risk of several institutions at the same time, like the study of Yun and Moon (2014). They claim that the multivariate GARCH models have an advantage in capturing the time-varying systemic risk exposure of a financial institution.

### 2.1 Turkish banking sector

The current regulation and supervision process of the banking system in Turkey was supported after the 2000–2001 crisis with the precautions taken for restructuring the sector, and the sector was empowered against the financial crises. Turkey faced two serious banking crises in November 2000 and February 2001. This led to more than 2 billion people lost their jobs. Furthermore, the government suspended the banking licenses of 22 banks, and spent USD 53.6 billion (almost 33% of the GPD of Turkey) for restructuring the banking sector. Before the crises, the sector focused on public finance instead of funding real sector, and the ratio of deposit to credit was very low. The banking sector operated low capital ratios, high leverages, maturity mismatch and a lack of risk management system for a long time. This led to the increase of the sector sensitivity to liquidity, interest rate and currency risks (Talasli, 2013).

After these two crises, the government started a solid banking rehabilitation program. The purpose of the program was to recover the private banks, to ease the resolution of the banks transferred to Saving Deposit Insurance Fund, to restructure public banks and constitute legal and institutional framework. The government introduced the limited deposit guarantee on July 2004 (Kibritcioglu, 2005). The legal framework and supervision of the Turkish banking sector was closely aligned with international standards like Basel II and EU standards with the completion of the recovery program. Turkish banks shown a rapid improvement and they started to work more efficiently and competitively under the regulatory control of the Banking Regulation and Supervision Agency (BRSA) and CBRT.

The last global crisis in 2008 had limited adverse effects on the banks operating in Turkey since they were high quality capital ratios compare to US and European banks, low liquidity risks, good management of mismatch, effective risk management system and solid public supervision. However, the sector imposed the negative effects of the last financial crisis through the decline in international trade of Turkish goods and services, decreasing external funds and the decline in credit volume (Talasli, 2013). The CBRT and BRSA took several actions to ease the negative effects of the last crisis on Turkish banks such as allowing the banks to reclassify the securities in their balance sheets.

When we look at the banking sector in Turkey, banks and their affiliates have dominated the Turkish financial sector for many decades. The financial sector reached an asset size of 915.56 million EUR by the end of 2016, and the banking sector had a share of 81%. With an asset size of 740 billion EUR by the end of 2016, the Turkish banking sector was ranked 13th among the banks in the European Union. As of the end of 2016, a total of 52 banks were operating in Turkey. Of these banks, 34 are deposit banks, 13 are development and investment banks and 5 are participation banks. There are 21 foreign-funded banks in the sector, which operate as a participation bank or as a branch office. Among the 13 development and investment banks, 3 are publicly funded, 6 are private and 4 are foreign-funded banks (The Banks Association of Turkey, hereafter known as TBB, 2016).

Regarding the capital structure of the Turkish banking sector, it has good capital quality, and the main capital accounts for more than 90% of the resources. While the minimum capital sufficiency ratio of 8% has been applied in the Turkish banking sector since 1992, the BRSA has asked the banks to meet the target ratio of 12% as of 2016. BRSA (2010) stated that these measures have empowered the Turkish banking sector even more, and Turkey became the only country among all OECD countries that did not need public capital support in the latest financial crisis. As of the end of 2016, the capital sufficiency ratio of the Turkish banking sector had reached 15.6% and the core capital ratio was 13.2%. This ratio is well above the 8% ratio required by Basel III (TBB, 2017). Similarly, the leverage ratio has been calculated in the Turkish banking sector for a long time. CBRT, BRSA and other relevant authorities closely monitor the credits of Turkish banks and impose limitations where necessary.

Many countries have implemented several regulations to prevent systemic risk. In Turkey, the Financial Stability Committee was established on June 3, 2011. The committee consists of CBRT, the Undersecretariat of Treasury, BRSA, the Capital Market Board and The Saving Deposit Insurance Bank (Basci, 2012). The Systemic Risk Assessment Group established on October 30, 2012, replaced the Systemic Risk Coordination Committee, which was founded in 2009. The aim of the group is that potential events that might cause systemic risk are detected in advance and necessary measures are taken and the coordination, cooperation and information sharing among the parties are facilitated. Furthermore, a Systemic Risk Assessment Technical Sub-Committee was established to support the technical activities of the group (BRSA, 2016).

The “Regulation on Systemically Important Banks” issued by BRSA was published in the Official Gazette, dated February 23, 2016. This regulation requires the utilization of the indicator-based method in the determination of systemically important banks and states that the quantity, complexity, interconnectedness and non-substitutability criteria and the indicators and sub-indicators suggested by BCBS and Financial Stability Board (FSB) shall be taken into consideration. This final regulation provides the required regulations on systemic risk based on the method suggested by the BCBS and FSB.

## 3 Data

Banks and their affiliates and their subsidiaries operating in Turkey constitute almost the entire Turkish financial sector. Therefore, the present study focuses on evaluating the spillover risk and identifying the systemic risk contributions of major Turkish banks for the period from January 2005 to December 2016. The data are composed of 13 big Turkish banks: 3 public, 1 state-development and 9 private banks, out of a total of 52 banks, which account for almost 81% of the total assets of the banking sector in Turkey and are therefore considered too-big-to-fail. Two public banks, Halkbank and Vakifbank, are publicly traded on the stock market, whereas Ziraat is not. In Turkey, public banks have an important role both socially and financially, and therefore, they are included in the study. The main criteria for choosing this set of banks are their systemic importance and publicly available data. An analysis involving these thirteen banks is believed to be able to give a general idea of the systemic risks of the Turkish banking sector. We restrict the study only to banks and do not include other financial institutions, e.g., insurance companies, etc. A list of banks and % share of assets in the Turkish banking sector are presented in Appendix 1.

The estimations are based on quarterly asset returns data of 13 banks and quarterly state variables covering the study period. For VaR and CoVaR, we need publicly traded institutions. However, stock market flotation of the Turkish financial sector was improved after 2004. For example, the initial public offerings of two public banks, Vakifbank and Halkbank, were held in 2005 and 2007, while many private banks were publicly opened before that time. Thus, we chose the study period from 2005 to 2016 based on public data availability.

***Measuring Losses:*** CoVaR estimation methodology was originally based on return losses on market equity of institutions and basically relies on publicly available data. Adrian and Brunnermeier (2009) used the market-valued total assets in their study because their idea was that the market value of assets is closely related to the supply of credit to the real economy. In the present study, the method and models from Adrian and Brunnermeier (2009) were used. The losses were computed based on market equity. The quarterly total asset returns of a financial institution i (X^i^) were computed using the following formula (Adrian and Brunnermeier, 2009):

(1) $$X^{i}=\frac{ME_{t}^{i}.LEV_{t}^{i}-ME_{t-1}^{i}.LEV_{t-1}^{i}}{ME_{t-1}^{i}.LEV_{t-1}^{i}}=\frac{A_{t}^{i}-A_{t-1}^{i}}{A_{t-1}^{i}}$$

(2) $$\;A_{t}^{i}=ME_{t}^{i}.LEV_{t}^{i}=BA_{t}^{i}.\left(\frac{ME_{t}^{i}}{BE_{t}^{i}}\right)$$

where ${\text{A}}_{\text{t}}^{\text{i}}$ and $A_{t-1}^{i}$ show the market value of the total assets of institution i at times t and (t-1), respectively; ${\text{BA}}_{\text{t}}^{\text{i}}$ and $BE_{t}^{i}$ shows the book value of total assets and total equity of institution i at time t, respectively; $ME_{t}^{i}$ represents the market value of total equity of institution i; and $LEV_{t}^{i}=BA_{t}^{i}/BE_{t}^{i}$ shows the leverage ratio of i at time t. The asset returns of each bank are converted to the form of logarithmic returns. The asset returns of the financial system are calculated from the weighted average returns of all these thirteen banks with the following formula:

(3) $$\sum_{i}\frac{A_{t}^{i}}{\sum_{j}A_{t}^{j}}X_{t}^{i}=\frac{A_{t}^{system}-A_{t-1}^{system}}{A_{t-1}^{system}}=X_{t}^{system},\;\;i\neq j$$

When Eq. (1) is reviewed, to calculate X^i^, the share unit values of the institution as valued in the market as well as its financial statements are required. However, it is only possible to obtain the share unit prices for publicly traded companies. Ziraat is not quoted in the national stock market, but it owns more than 14% of total assets of the banking sector in Turkey, so it has overwhelmingly dominated the Turkish financial sector. Hence, including Ziraat in the study was important. Because Ziraat is not publicly traded, the price per share has not been obtained and was calculated instead based on the "XBANK-Banking Index". In the existing literature, XBANK has been used as a proxy for the Turkish financial sector because of providing more accurate and reliable results. Cepni et al. (2019) examine five developing markets, including Brazil, Indonesia, Mexico, South Africa and Turkey. They use XBANK as a financial indicator for Turkey in their study. Chadwick and Ozturk (2018) use XBANK as the most common variable that represent banking stress in order to construct a single financial stress indicator for Turkey. Sener et al. (2019) use XBANK in order to examine the Turkish financial market in their study. XBANK, an indicator for the banking sector in Turkey, was used to calculate the price per share of Ziraat. The returns were calculated using this index over the 2nd session closing values at the end of each quarter. It was assumed that the number of shares is equal to the equity amount and that the price per share of Ziraat moves in the same direction as the XBANK index. Based on these assumptions, the market value of total equity was obtained by multiplying the book value of total equity with the XBANK percentage return. In the following step, the asset returns of Ziraat were calculated by using Eq. (1).

Since Halkbank was offered to the public in May 2007, the price of the first shares in the study was relevant to the date of June 30, 2007. There is no share price quotation for the nine dates covering the date range from March 31, 2005, to March 31, 2007. For Halkbank, the asset returns for these nine dates were also obtained using the method used for Ziraat. Vakifbank's initial public offering was held on August 25, 2005. The initial share price for Vakifbank in the study was on the date of December 31, 2005. There is no share price quotation related to the following dates: March 31, 2005, June 30, 2005, and September 30, 2005. For Vakifbank, the asset returns for these three dates were obtained by using the method used for Ziraat. TEB voluntarily withdrew from the stock market listing on June 25, 2015. TEB does not have any share price quotation since then, including the date of June 30, 2005. The most recent share price in the study is from March 31, 2015. For these seven TEB dates, the same method was used for obtaining its return.

***State Variables:*** We later added state variables M_t_ to the model for capturing time variation in asset returns. We expect that the state variables might have different effects on the Turkish banks included in the study. To keep the data manageable, the study is restricted to a small set of risk factors. The state variables used in the study are classified into two groups: macroeconomic variables and bank variables demonstrating an individual bank characteristic. The definitions of the variables and sources are listed in Appendix 2.

Two macroeconomic variables, which are the return of BIST100 (hereafter *BIST100*) and the return of equity volatility (hereafter *Volatility*), are added to the model as predictors. These macro variables are selected based on the assumption that these factors might affect the asset returns of banks. The two macrovariables reflect investor approach and their expectations, and they are used as a proxy for the financial market. For example, Gunay (2017) examines different value-at-risk models in order to measure downside investment risk under different methods. The author selects BIST100 as one of the variables since BIST100 includes a wide variety of asset risks such as stocks, currency rates and commodity. Aydin and Cavdar (2015) investigate the relationship between the exchange rate of US Dollar-Turkish Lira (USD/TRY), gold prices and the Borsa Istanbul (BIST) 100 index. In this study, BIST100 index is used as an indicator for the Turkish financial market as in the other studies such as Hatipoglu and Umut (2019), Chadwick and Ozturk (2018) and Sengul and Yilmaz (2019). In the literature, it is seen that the equity volatility is chosen as a common variable in many researches on systemic risk. Adrian and Brunnermeier (2009) select a set of state variables for measuring systemic risk based on capturing time variation, being liquid and tractable, and equity volatility is one of them. Zeb and Rashid (2015) aim to specify the domestic systemically important banks of Pakistan using the dataset of 21 commercial banks. They use equity volatility as one of the state variables for estimating its effect on different banks of Pakistan. Moreover, Talasli (2013) use annualized volatility of stock returns as a variable which is calculated as square root of 250 times standard deviation of daily stock returns, see also Akkoyun et al. (2013) who calculate yearly volatility of equity by annualizing the daily volatility of the most recent 21 trading days.

With respect to bank variables, the last financial crisis in 2008 demonstrated the importance of capital requirements of financial institutions to mitigate systemic risk adverse effects since they can be used for enhancing financing stability. The effect of capital has captured considerable attention in the global financial market, and it is accepted to be associated with systemic risk. The state variables of this part are selected closely related to capital on the bank level except for the liquid assets ratio since it is directly related to the capacity of a bank to remain liquid in order to pay short term debt. The variable of liquidity can be used as a signal of possible future capital needs. We focus on the bank-level balance sheet characteristics to examine the relationship with systemic risk. Since the risks carried on the banks’ balance sheets are different, these differences should be considered. *Tier 1 Capital Ratio* (hereafter *Tier 1*)*, Capital Adequacy Ratio* (hereafter *CAR*)*, Leverage Ratio* (hereafter *Leverage*) and *Liquid Assets Ratio* (hereafter *Liquidity*) are added to the model as individual bank variables. In the existing literature, these variables are selected for measuring the systemic risk of financial institutions in terms of capital issue. For example, Laeven et al. (2014) research how bank characteristics affect bank risk during the crisis, and they use the tier 1 capital ratio and a leverage ratio for measuring bank capitalization, see also the studies of Demirguc-Kunt et al. (2013), Anginer and Demirguc-Kunt (2014), Girardi and Ergün (2013), Bernal et al. (2014).

## 4 CoVaR methodology

The CoVaR method was first proposed by Adrian and Brunnermeier (2009). They defined CoVaR as the VaR of the whole financial system conditional on a financial institution being in a particular situation, such as experiencing difficulties or everything being in order. When we discuss an institution facing difficulties, it generally means that the stock returns of this institution approach its VaR level. CoVaR allows us to measure the negative externalities and spillover risk. Thus, a larger CoVaR means that an institution i imposes a larger negative externality on another institution j when the returns of i is at its VaR level.

VaR^i^_q_ is implicitly defined as the q quantile, i.e.,

(4) $$Pr\left(X^{i}\leq VaR_{q}^{i}\right)=q\%$$

where X^i^ denotes the (return) loss of institution i (hereafter called the asset returns) for which the VaR^i^_q_ is defined (Adrian and Brunnermeier, 2009). X usually refers to a crisis situation for i, which approaches the VaR value with the possibility of q%. The VaR value is usually a negative number, but its sign is generally switched.

CoVaR^j/i^ is defined as the VaR of an institution j conditional on the return of another institution i being at its VaR level, i.e., $X^{i}=VaR_{q}^{i}$. That is, $CoVaR_{q}^{j/i}$ is only the conditional quantile distribution and denoted as follows:

(5) $$\text{Pr}\left(X^{j}\leq CoVaR_{q}^{j/i}|X^{i}=VaR_{q}^{i}\right)=q\%$$

The systemic risk contribution of a particular institution i to the institution j is denoted by ΔCoVaR:

(6) $$∆CoVaR_{q}^{j/i}=CoVaR_{q}^{j/X^{i}=VaR_{q}^{i}}-CoVaR_{q}^{j/X^{i}=Median^{i}}\text{, }Median^{i}=VaR_{50}^{i}$$

ΔCoVaR^j/i^_q_ represents the marginal systemic risk contribution of i to the value-at-risk of j relative to its median state. If j is a financial system, ΔCoVaR is rewritten as follows and estimated for each institution, respectively.

(7) $$∆CoVaR_{q}^{system/i}=CoVaR_{q}^{system/X^{i}=VaR_{q}^{i}}-CoVaR_{q}^{system/X^{i}=VaR_{50}^{i}}$$

$∆CoVaR_{q}^{system/i}$ allows us to determine how much the change in the VaR value of the financial system conditional on the asset returns of an institution i moves per unit from the median state to its VaR level for the q quantile. We can also compute $∆CoVaR_{q}^{i/system}$, which captures the vulnerabilities of an institution i when the system gets into distress. This paper focuses on estimating the contribution of an individual institution to systemic risk of the whole financial system using Eq. (7).

### 4.1 Estimation method of CoVaR: quantile regression

We use quantile regression to estimate CoVaR. Although quantile regression is not the only way to measure CoVaR, it is the most preferred and a particularly efficient method. Quantile regression, which was first proposed by Koenker and Bassett (1978), was developed for estimating conditional quantile functions. In quantile regression models, the relation between a predictor variable(s) and the response variable is searched in a proper quantile(s) (Koenker, 2005). This approach enables us to make comparisons of different quantiles in which the response variable can be affected by the predictors in different ways. In quantile regression, there is no need to make a distribution assumption. Quantile regression estimates the given certain values of variables by using conditional median or any other quantile of the dependent variable. In this study, the bootstrapping method was used for estimating the standard deviation of the quantile regression. Here, CoVaR is estimated under a conditional and unconditional framework. In the unconditional case, CoVaR is accepted to be constant over time, but for capturing time variation, CoVaR is estimated as a function of state variables.

#### 4.1.1 Unconditional CoVaR estimation

In the unconditional CoVaR method, CoVaR does not change over time. We regress the asset returns of each bank as an independent variable to the return of the financial system as a dependent variable in the quantile regression model. Eq. (8) represents the quantile regression model of the asset returns of a financial institution i for the q-quantile. The predicted value of this equation gives us the computed VaR value of an institution i. Similarly, the model is constructed for the system, i.e., X^system^, and its prediction value equals to computed VaR value, i.e., VaR^system^.

(8) $$X_{q}^{i}=\alpha_{q}^{i}+\varepsilon_{q}^{i}$$

To obtain the quantile estimation of CoVaR, the model is set up as in follows:

(9) $$X^{j}=\alpha_{q}^{i}+\beta_{q}^{i}X^{i}+\varepsilon$$

where α_q_ represents the intercept for a specified q quantile when X^i^ is equal to zero. β_q_ represents the change in the asset returns of j when there is a per-unit change in X^i^ at the same quantile. The predicted value of the system return conditional on the losses of an institution i for the q quantile is given by

(10) $${\hat{X}}_{q}^{system/X^{i}}={\hat{\alpha }}_{q}^{i}+{\hat{\beta }}_{q}^{i}X^{i}$$

According to the definition of VaR, it is concluded that

(11) $$CoVaR_{q}^{system/X^{i}}=VaR_{q}^{system}|X^{i}={\hat{X}}_{q}^{system/X^{i}}$$

When we use VaR^i^_q_ as the predicted value of X^i^, i.e., X^i^ = VaR^i^_q_, and use the predicted values of the α and β coefficients, the $CoVaR^{system/X^{i}}$can be deduced as in (12).

(12) $$CoVaR_{q}^{system/X^{i}=VaR_{q}^{i}}=VaR_{q}^{system/X^{i}=VaR_{q}^{i}}={\hat{\alpha }}_{q}^{i}+{\hat{\beta }}_{q}^{i}VaR_{q}^{i}$$

If we take X^i^ = VaR^i^_50_, the above expression is shown as follows:

(13) $$CoVaR_{q}^{system/X^{i}=VaR_{50}^{i}}={\hat{\alpha }}_{q}^{i}+{\hat{\beta }}_{q}^{i}VaR_{50}^{i}$$

The systemic risk contribution of institution i to the VaR of the whole financial system is computed with ΔCoVaR given by

(14) $$∆CoVaR_{q}^{system/i}={\hat{\beta }}_{q}^{system/\text{i}}\left(VaR_{q}^{i}-VaR_{50}^{i}\right)$$

***Hypothesis:*** Based on the theoretical explanation above, the hypothesis of this part can be formed as follows:

H1 The quarterly VaR of the asset returns of the banks or the quarterly losses of the banks have a positive significant relationship with the quarterly system returns or the system losses, and thus any increase in the VaR of the returns of the banks is associated with high contribution to the conditional VaR of the system and thereby systemic risk.

#### 4.1.2 CoVaR estimation for capturing time variation

To capture time variation in the joint distribution of X^system^ and X^i^, VaR and CoVaR are estimated as a function of state variables. In this part, the extent to which the value at risk of the financial system changes was measured by adding different independent variables to the CoVaR model in addition to the asset returns of institution i. Macroeconomic variables or individual bank variables can be added as predictors. After adding these predictors to the model, the CoVaR of each institution is computed again. We indicate time-varying with the subscript t. We run the following quantile regression models, which differ from the model of Adrian and Brunnermeier (2009) as we use M_t_ instead of lagged variables, i.e., M_t-1_, since the number of observations in this study is rather small (48 observations for each bank), whereas if we were to use lagged variables, it could cause a substantial loss of information.

(15) $$X_{t}^{i}=\alpha_{q}^{i}+\delta_{q}^{i}M_{t}+\varepsilon_{q,t}^{i}$$

(16) $$X_{t}^{system/i}=\alpha_{q}^{system/i}+\delta_{q}^{system/i}M_{t}+\gamma_{q}^{system/i}X_{t}^{i}+\varepsilon_{q,t}^{system/i}$$

To obtain VaR^i^_q,t_ and CoVaR^i^_q,t_; we generate the estimated values of the parameters from Eqs. (15) and (16), and based on these estimations, we will compute the individual VaR values and CoVaR as well, given as follows:

(17) $$VaR_{q,t}^{i}={\hat{\alpha }}_{q}^{i}+{\hat{\delta }}_{q}^{i}M_{t}$$

(18) $$CoVaR_{q,t}^{i}={\hat{\alpha }}_{q}^{system/i}+{\hat{\delta }}_{q}^{system/i}M_{t}+{\hat{\gamma }}_{q}^{system/i}VaR_{q,t}^{i}$$

where M_t_ represents the M-vector of independent variables; δ_q_ represents the change in the asset returns of an institution i when there is a per-unit change in M_t_; γ_q_ represents the coefficients that show the change in the asset returns of the system when there is a per-unit change in X^i^ for the q quantile. Finally, the systemic risk contribution of each institution to the financial system, i.e., ΔCoVaR is estimated:

(19) $$∆CoVaR_{q,t}^{system/i}={\hat{\beta }}_{q}^{system/\text{i}}\left(VaR_{q,t}^{i}-VaR_{50,t}^{i}\right)$$

***Hypotheses:*** For time-varying estimation of the CoVaR model, we add macroeconomic variables and bank-specific variables into the model. With respect to the macroeconomic variables, the returns of BIST100 index are expected to have a positive impact on the VaR of the financial system returns. Furthermore, we expect that higher volatility levels increase the VaR of the system returns. Thus, the hypotheses of this part are as follows:

H2 The quarterly BIST100 has a positive significant relationship with the quarterly VaR of the system returns, and thus any increase in the VaR of the BIST100 is associated with high contribution to the conditional VaR of the system and thereby systemic risk.

H3 The quarterly volatility has a positive significant relationship with the quarterly VaR of the system returns, and thus any increase in the VaR of the volatility is associated with high contribution to the conditional VaR of the system and thereby systemic risk.

According to bank variables, leverage measured by total capital divided by total assets are expected to have a negative impact on the VaR of the financial system returns. That is, when the leverage of a bank is decreased, the VaR estimation is increased, and the capital shortage of the bank might be increased. Furthermore, we also expect liquidity to have a negative effect on the VaR of the returns of the system. To examine the relationship between capital ratios and systemic risk, two main capital ratios, Tier 1 and CAR, are selected. We expect a negative relation between two capital ratios and the VaR of the system. Considering the above information, we form the following hypotheses:

H4 The quarterly leverage ratio is negatively related to the quarterly VaR of the system returns, and thus low leverage is associated with high contribution to the conditional VaR of the system and systemic risk, as well.

H5 The quarterly liquid assets ratio is negatively related to the quarterly VaR of the system returns, and thus low liquid assets ratio increase the conditional VaR of the system and thereby systemic risk.

H6 The quarterly tier 1 capital ratio has a negative significant relationship with the quarterly VaR of the system returns, and thus low tier 1 capital ratio increase the conditional VaR of the system and increase systemic risk.

H7 The quarterly capital adequacy ratio is negatively significant relationship with the quarterly VaR of the system returns, and thus low capital adequacy ratio increase the conditional VaR of the system and thereby systemic risk.

## 5 Empirical results

We present the estimation results of VaR, CoVaR and ΔCoVaR for each bank using quantile regression with the confidence level of q = 5%. To avoid confusion, the results section is structured as follows: First, descriptive statistics of the asset returns are presented. Second, only the asset returns of thirteen banks are taken into consideration; the contribution of each bank to the systemic risk of the financial system is estimated, and the results are presented. Since CoVaR is unconditional, it is accepted that CoVaR estimations are constant over time during the period 2005–2016. Then, the time-varying estimation results based on state variables are presented in the third and fourth stages. That is, two macroeconomic variables and four bank variables are included in the model as predictors, and conditional estimation results are presented and discussed. The conditional estimation with state variables allows us to survey how risk measures, i.e., VaR, CoVaR and ΔCoVaR, change over time. Finally, the table demonstrating VaR, CoVaR and ΔCoVaR estimation results of each bank and their ranking is presented and discussed.

### 5.1 Summary statistics

The descriptive statistics for the asset return of each bank and the financial system covering the period from 2005 to 2016 are shown in Table 1. According to the table, Halkbank (9%) and Vakifbank (8%) have the highest average return for quarterly periods. Another way to express the risk is through volatility calculated as the standard deviation of returns. Halkbank and Vakifbank are two public banks with the highest volatilities. As these two banks have the highest returns, they have the highest standard deviation values, which is an expected result. The lowest volatility belongs to Akbank. According to the kurtosis and skewness values, we can conclude that the asset returns are not normally distributed since these two values are differ from 3 and 0, and especially certain banks have larger kurtosis values.

**Table 1:** Descriptive statistics of the asset returns.

	Mean	Stan.Dev.	Median	Maximum	Minimum	Skewness	Kurtosis
Ziraat	0.04	0.22	0.00	0.56	-0.44	0.31	2.63
Halkbank	0.09	0.35	0.05	2.07	-0.40	3.80	22.56
Vakifbank	0.08	0.35	0.05	1.81	-0.62	2.19	13.71
Isbank	0.04	0.18	0.04	0.35	-0.36	-0.15	2.60
Akbank	0.04	0.18	0.04	0.47	-0.32	-0.04	2.82
Garanti	0.06	0.21	0.07	0.56	-0.74	-1.11	6.44
Yapi Kredi	0.04	0.23	0.09	0.58	-0.90	-1.46	7.97
Denizbank	0.07	0.25	0.03	0.79	-0.49	0.66	4.12
Finansbank	0.06	0.21	0.02	0.66	-0.53	0.17	4.19
TEB	0.02	0.40	0.05	0.77	-1.77	-1.85	9.65
Sekerbank	0.02	0.25	0.03	0.55	-0.60	-0.47	3.58
TSKB	0.05	0.26	0.06	0.87	-0.70	0.34	4.99
Kalkinma	0.04	0.24	0.03	0.58	-0.61	-0.21	4.02
System	0.08	0.18	0.08	0.59	-0.35	0.42	4.18

### 5.2 Unconditional CoVaR estimation results

The unconditional CoVaR estimation allows us to examine how the VaR of the system returns are affected when a bank's return is on its VaR level. The CoVaR model was established as follows:

(20) $$\text{log}\left(X_{q}^{system/i}\right)=\alpha_{q}^{system/i}+\beta_{q}^{system/i}\text{log}\left(X^{i}\right)+\varepsilon_{q}^{system/i}$$

The CoVaR model parameters for the 5^th^ quantile of each bank are estimated by using quantile regression. The t-test is applied for the significance of the coefficients estimated from the CoVaR model, and the parameters are found to be significant for the banks other than Denizbank, Finansbank and Kalkinma. The hypothesis is not rejected for all the banks except for these three banks. The results are presented in Table 2. This table reveals that there is a high interconnection between the asset returns of individual banks and the financial system. The coefficient for the individual bank's asset returns, i.e., $\beta_{q}^{\;\;system/i}$, is positive for all the banks, and it is significant for ten banks, especially for Isbank, Garanti, Akbank and Yapi Kredi, with a high t-statistic. The positive coefficient implies that the financial system would incur larger losses when an individual financial institution falls into distress. Thus, the positive β estimation seems to provide strong evidence of spillover effects between the asset returns of the banks and the financial system (see Table 2).

**Table 2:** Regression parameters of the unconditional CoVaR model.

Model: (CoVaR5%): $\text{log}X_{q}^{system/i}=\alpha_{q}^{system/i}+\beta_{q}^{system/i}\text{log}X^{i}+\varepsilon_{q}^{system/i}$			
(logX^system^)	α^i^	β_i_	Pseudo R^2^
Ziraat	-0.12•	0.54∗∗	0.22
	(-1.68)	(2.72)	
Halkbank	-0.15∗∗	0.35∗	0.37
	(-3.32)	(2.27)	
Vakifbank	-0.10∗	0.38∗∗	0.48
	(-2.43)	(2.79)	
Isbank	-0.064∗∗∗	0.86∗∗∗	0.62
	(-4.12)	(10.74)	
Akbank	-0.08∗∗∗	0.81∗∗∗	0.64
	(-4.46)	(11.09)	
Garanti	-0.13∗∗	0.74∗∗∗	0.51
	(-3.34)	(5.56)	
Yapi Kredi	-0.08∗	0.80∗∗∗	0.44
	(-2.13)	(4.23)	
Denizbank	-0.22∗∗∗	0.19	0.12
	(-4.12)	(1.34)	
Finansbank	-0.21∗∗	0.19	0.06
	(-3.36)	(0.70)	
TEB	-0.12∗∗∗	0.366∗∗∗	0.37
	(-4.39)	(3.77)	
Sekerbank	-0.12∗∗	0.38∗	0.31
	(-3.11)	(2.12)	
TSKB	-0.22∗∗∗	0.41∗	0.20
	(-4.27)	(2.69)	
Kalkinma	-0.15∗	0.14	0.01
	(-2.11)	(0.55)	

*Notes:* This table reports the coefficients of the CoVaR model with t-statistics which are reported in the parantheses. In the table, the system returns, i.e., logX^system^, in bold on the top left hand side is the dependent variable, regressed on the independent variable which is the asset returns of the bank labelled β_i_ in the rows. Thus, the first row represents the VaR of the system condition on the asset returns of Ziraat being at its VaR level for q = 5%.

(∗∗∗) 0.001 (∗∗) 0.01 (∗) 0.05 and (•) 0.10.

### 5.3 Estimation results of CoVaR based on macroeconomic variables

In this part, the model generated for the asset returns of each bank is written as follows:

(21) $$\text{log}\left(X_{t}^{i}\right)=\alpha_{q}^{i}+\beta_{1_{q}}^{i}logBIST100_{t}+\beta_{2_{q}}^{i}logVolatility_{t}+\varepsilon_{q,t}^{i}$$

The estimates of the α and β coefficients from the above model give us a conditional VaR series of an individual bank, i.e., VaR^i^_q,t_. Furthermore, with the estimation of the coefficients for α and β_i_ from the following model, we construct CoVaR^system/i^_q,t_.

(22) $$\;\text{log}\left(X_{t}^{system/i}\right)=\alpha_{q}^{system/i}+\beta_{1_{q}}^{system/i}logBIST100_{t}+\beta_{2_{q}}^{system/i}logVolatility_{t}+\;\beta_{3_{q}}^{system/i}\text{log}\left(X_{t}^{i}\right)+\varepsilon_{q,t}^{system/i}$$

First, the parameters of the individual VaR model of the financial system for the 5^th^ quantile based on Eq. (21) are estimated in order to examine how the VaR of the system is affected by per unit change in macroeconomic variables in this quantile. A t-test was applied to analyse the significance of the coefficients for the financial system, and the results are listed in Table 3. According to the results, the coefficient of *BIST100* for the system was estimated as 1.21 for the 5^th^ quantile, meaning that the per unit change in *BIST100* would increase the asset returns of the system by 1.21. Moreover, the coefficient of *BIST100* was significant and positive, whereas the coefficient of *Volatility* was insignificant.

**Table 3:** Regression parameters of the VaR model for the system based on macro variables.

Model: (VaR5%): logX^system^ = α + β_1_ (logBIST100) + β_2_ (logVolatility) + Ɛ				
(logX^system^)	α	β_1_	β_2_	Pseudo R^2^
q = 5. Quantile	-0.06∗	1.21∗∗∗	0.12	0.63
	(-2.30)	(9.70)	(1.43)	

(∗∗∗) 0.001 (∗∗) 0.01 (∗) 0.05 and (•) 0.10.

The CoVaR estimation results are presented in Table 4. It was concluded that the coefficients of the asset returns $\beta_{3}^{\;\;}\left(logX^{i}\right)$ for Halkbank, Vakifbank, Isbank, Akbank, Garanti, Denizbank and TEB were positive and significant on the returns of the financial system. For Ziraat, its coefficient affected the system return negatively. The beta coefficient for BIST100 is positive for all the banks and strongly significant on the system return, especially for Garanti, Ziraat, Halkbank, Denizbank, Finansbank, TSKB and TEB. This result implies that the first hypothesis is not rejected. For volatility, its coefficient is significant only for Akbank, Garanti and TSKB, and thus, we can conclude that volatility could not be significant with the system (see Table 4).

**Table 4:** Regression parameters of the CoVaR model based on macro variables.

Model: (CoVaR5%): log (X^system/i^) = α^i^ + β_1_ (logBIST100) + β_2_ (logVolatility) + β_3_ (logX^i^) + Ɛ					
(logX^system^)	α^i^	β_1_	β_2_	β_3_	Pseudo R^2^
Ziraat	-0.04∗∗	1.27∗∗∗	0.04	-0.22∗	0.68
	(-2.83)	(7.74)	(0.62)	(-2.04)	
Halkbank	-0.06∗∗	0.63∗∗	0.09	0.28∗	0.65
	(-2.96)	(3.42)	(1.22)	(2.26)	
Vakifbank	-0.05∗∗	0.57∗∗	0.04	0.29∗	0.68
	(-3.38)	(2.88)	(0.83)	(2.25)	
Isbank	-0.05∗	0.62∗∗	0.10	0.39∗	0.66
	(-2.58)	(3.21)	(1.34)	(2.63)	
Akbank	-0.01∗∗	0.47∗	0.05•	0.56∗∗	0.73
	(-2.91)	(2.38)	(1.70)	(2.99)	
Garanti	-0.02∗∗∗	0.68∗∗∗	-0.04•	0.24∗∗∗	0.74
	(-4.66)	(8.39)	(-1.74)	(4.82)	
Yapi Kredi	-0.07∗	0.74∗∗	0.13	0.23	0.61
	(-2.65)	(3.11)	(1.46)	(1.47)	
Denizbank	-0.07∗	0.95∗∗∗	0.09	0.15•	0.63
	(-2.59)	(6.58)	(1.12)	(1.69)	
Finansbank	-0.06∗	1.01∗∗∗	0.10	0.13	0.64
	(-2.36)	(7.73)	(1.06)	(1.38)	
TEB	-0.06∗∗	1.01∗∗∗	0.09	0.10•	0.66
	(-2.96)	(7.89)	(0.97)	(1.79)	
Sekerbank	-0.07∗∗	1.08∗∗∗	0.11	-0.07	0.63
	(-2.84)	(6.55)	(1.34)	(-0.65)	
TSKB	-0.07∗∗	1.27∗∗∗	0.13•	-0.06	0.64
	(-2.96)	(8.89)	(1.77)	(-0.64)	
Kalkinma	-0.05∗	1.09∗∗∗	0.08	0.07	0.65
	(-2.11)	(6.20)	(0.97)	(0.80)	

*Notes:* This table reports the coefficients of the CoVaR model based on macroeconomic variables. t-statistics are reported in the parantheses. In the table, the system returns, i.e., logX^system^, in bold on the top left hand side is the dependent variable, regressed on logBIST100 labelled β_1_ and logVolatility labelled β_2_ in addition to the asset returns of the bank labelled β_3_ in the rows. Thus, the first row represents the VaR of the system condition on the macroeconomic variables and the asset returns of Ziraat being at its VaR level for q = 5%. (∗∗∗) 0.001 (∗∗) 0.01 (∗) 0.05 and (•) 0.10.

### 5.4 Estimation results of CoVaR based on individual bank variables

The model of this part generated for each bank separately is as follows:

(23) $$\;\text{log}\left(X_{t}^{i}\right)=\alpha_{q}^{i}+\beta_{1_{q}}^{i}Tier1_{i,t}+\beta_{2_{q}}^{i}CAR_{i,t}+\beta_{3_{q}}^{i}Liquidity_{i,t}+\beta_{4_{q}}^{i}Leverage_{i,t}+\varepsilon_{q,t}^{i}$$

The model for the financial system is constructed in a similar way. The bank variables of the financial system are formed by taking the average of the individual bank variables of thirteen banks. The model generated for measuring the effect of bank-i with its bank variables on the system is as follows:

(24) $$\;\text{log}\left(X_{t}^{system/i}\right)=\alpha_{q}^{system/i}+\beta_{1_{q}}^{system/i}Tier1_{i,t}+\beta_{2_{q}}^{system/i}CAR_{i,t}+\beta_{3_{q}}^{system/i}Liquidity_{i,t}+\;\beta_{4_{q}}^{system/i}Leverage_{i,t}+\beta_{5_{q}}^{system/i}\text{log}\left(X_{t}^{i}\right)+\varepsilon_{q,t}^{system/i}$$

With the estimates of the α and β coefficients from above model, CoVaR^system/i^_q,t_ is given by

(25) $$CoVaR_{q,t}^{system/i}={\hat{\alpha }}_{q}^{system/i}+{\hat{\beta }}_{1_{q}}^{system/i}Tier1_{i,t}+{\hat{\beta }}_{2_{q}}^{system/i}CAR_{i,t}+{\hat{\beta }}_{3_{q}}^{system/i}Liquidity_{i,t}+{\hat{\beta }}_{4_{q}}^{system/i}Leverage_{i,t}+{\hat{\beta }}_{5_{q}}^{system/i}VaR_{q,t}^{i}$$

We begin by examining the VaR5% estimations for the system. The VaR results for the financial system for the 5^th^ quantile based on Eq. (23) are presented in Table 5. According to the results, although the coefficients of the variables “*Tier 1*” and “*CAR*” are significant for the 5^th^ quantile, the coefficients of the “*Liquidity*” and “*Leverage*” are negative and insignificant for this quantile. Furthermore, the impact of Tier 1 on the VaR of the system is negatively significant, indicating that higher level of Tier 1 capital lead to lower risk. However, the opposite is true for CAR.

**Table 5:** Regression parameters of the VaR model for the system based on bank variables.

Model: (VaR5%): logX^system^ = α + β_1_(Tier1_system_) + β_2_(CAR_system_) + β_3_(Liquidity_system_) + β_4_ (Leverage_system_) + ϵ					
(logX^system^)	α	β_1_	β_2_	β_3_	β_4_
q = 5. Quantile	-0.39	-20.13∗	22.56∗∗	-0.80	-2.57
	(-0.67)	(-2.63)	(2.92)	(-0.39)	(-0.55)

(∗∗∗) 0.001 (∗∗) 0.01 (∗) 0.05 and (•) 0.10.

The results of the CoVaR model are given in Table 6. The findings show that the coefficient of (*logX*^*i*^_*t*_) is positive and significant for all the banks, except for Denizbank, Finansbank and Kalkinma. This value is strongly significant, especially for Akbank, Garanti, Yapi Kredi and Isbank, with high t-statistics; that is, the returns of the banks affect the financial system VaR in a positive way. Tier 1 is significant for Halkbank, Garanti, Yapi Kredi, Denizbank, Sekerbank and Kalkinma and has a negative value for 8 banks out of 13; that is, when Tier 1 capital might decrease for these eight banks, the conditional VaR of the system might increase. However, CAR is positive and significant for Halkbank, Isbank, Denizbank and Kalkinma, and negatively significant for Garanti. As expected, Tier 1 negatively affects the CoVaR in general, so we do not reject the hypothesis, but we do reject the hypothesis for CAR. Similar results are found in the study of Anginer and Demirguc-Kunt (2014). They examined the empirical relationship between bank capital and systemic risk using alternative definitions of capital ratios, including Tier 1 capital ratio. They conclude that higher levels of Tier 1 capital reduce systemic risk.

**Table 6:** Regression parameters of the CoVaR model based on bank variables.

Model: (CoVaR5%): log (X^system/i^) = α^i^ + β_1_(Tier1_i_) + β_2_(CAR_i_) + β_3_(Liquidity_i_) + β_4_(Leverage_i_) +β_5_(logX^i^) + Ɛ							
(logX^system^)	α^i^	β_1_	β_2_(CAR_i_)	β_3_(Liquidity_i_)	β_4_ (Leverage_i_)	β_5_ (logX^i^)	Pseudo R^2^
Ziraat	-0.29	-1.38	1.53	-0.49	3.71•	0.32∗∗∗	0.57
	(-1.23)	(-0.44)	(0.49)	(-1.28)	(1.75)	(3.82)	
Halkbank	0.38	-17.35∗∗	19.36∗∗	0.33	-9.22•	0.34∗∗	0.65
	(1.02)	(-3.12)	(3.13)	(0.58)	(-1.80)	(2.75)	
Vakifbank	0.30	3.41	-0.57	-1.67	-2.41	0.41∗∗	0.56
	(0.54)	(0.77)	(-0.16)	(-1.42)	(-0.48)	(2.90)	
Isbank	0.33	-0.78	3.01•	-0.73	-4.58	0.69∗∗∗	0.68
	(0.75)	(-0.22)	(1.68)	(-0.66)	(-1.09)	(4.96)	
Akbank	-0.27•	-0.48	1.28	-0.42	1.73	0.83∗∗∗	0.71
	(-1.89)	(-0.15)	(0.37)	(-1.23)	(1.10)	(7.60)	
Garanti	-0.48	7.54•	-8.27•	0.08	5.38•	0.92∗∗∗	0.69
	(-2.84)	(1.82)	(-1.78)	(0.17)	(2.01)	(6.98)	
Yapi Kredi	-0.34	7.44•	-5.25	1.11•	-0.51	0.77∗∗∗	0.58
	(-1.22)	(1.85)	(-1.34)	(1.94)	(-0.19)	(5.04)	
Denizbank	-2.60∗	-12.89•	20.34∗	3.66∗	0.26	0.19	0.27
	(-2.66)	(-1.69)	(2.14)	(2.45)	(0.02)	(0.69)	
Finansbank	-0.68	1.44	6.26	1.60	-6.26	-0.16	0.28
	(-1.18)	(-0.20)	(1.15)	(1.26)	(-1.08)	(-0.49)	
TEB	0.37	4.70	0.55	-1.81•	-6.01•	0.43∗∗∗	0.50
	(0.78)	(1.09)	(0.14)	(-1.75)	(-1.73)	(5.28)	
Sekerbank	-0.40	9.30∗	-1.43	-2.20∗∗∗	-1.35	0.53∗∗∗	0.46
	(-0.88)	(2.49)	(-0.26)	(-3.62)	(-0.49)	(3.70)	
TSKB	-0.89∗	-3.74	2.22	1.30	4.27•	0.49∗	0.41
	(-2.10)	(-0.64)	(0.40)	(0.87)	(1.78)	(2.22)	
Kalkinma	-0.01	-5.92•	6.05•	-0.76	-0.26	-0.08	0.37
	(-0.14)	(-1.72)	(1.72)	(-1.46)	(-0.54)	(-0.39)	

*Notes:* This table reports the coefficients of the CoVaR model based on individual bank variables. t-statistics are reported in the parantheses. In the table, the system returns, i.e., logX^system^, in bold on the top left hand side is the dependent variable, regressed on the independent variables which are Tier 1 capital ratio labeled β_1_, capital adequacy ratio labeled β_2_, liquid assets ratio labeled β_3_ and leverage ratio labeled β_4_ in addition to the asset returns of the bank_i,_ labeled β_5_ in the rows. Thus, the first row represents the VaR of the system condition on the bank variables and the asset returns of Ziraat being at its VaR level for q = 5%. (∗∗∗) 0.001 (∗∗) 0.01 (∗) 0.05 and (•) 0.10.

We observe that liquidity is significant only for Yapi Kredi, Denizbank, TEB and Sekerbank. However, leverage appears significant for Ziraat, Halkbank, Garanti, TEB and TSKB. This result suggests that we cannot reject the hypothesis of liquidity for certain banks such as TEB, Sekerbank, Isbank, Akbank, but can reject it for other banks such as Yapi Kredi and Denizbank. This case is also valid for leverage.

In conclusion, leverage and liquidity are not statistically significant for all the banks. However, leverage appears positive and significant especially for the largest banks such as Ziraat and Garanti, and negatively significant for Halkbank. One possible interpretation is that Ziraat and Garanti are well capitalized than smaller banks. This result is not suprising for Ziraat since Ziraat has the huge amount of public deposit, which helps to strength the capital structure of this bank. Similar results are found in the study of Borri et al. (2012). They find that leverage ratio is not statistically significant for the European banking sector. However, Girardi and Ergün (2013) reach different results. They find that leverage is important in explaining institutions’ contributions to systemic risk.

### 5.5 VaR, CoVaR and ΔCoVaR estimation results

Table 7 presents the results of VaR, CoVaR and ΔCoVaR based on the asset returns of the banks, macroeconomic variables and individual bank variables. We rank the banks with respect to their average VaR, CoVaR and ΔCoVaR values. The results can be summarized as follows.

i The unconditional CoVaR estimation results is reviewed, and we deduce the following:

**Table 7:** VaR, CoVaR and ΔCoVaR estimation results.

Banks	VaR5%	Rank	CoVaR5%	Rank	ΔCoVaR5%	Rank
	(Average)		(Average)		(Average)	
Ziraat						
Unconditional	-25.4	9	-25.24	10	-13.71	8
Macroeconomic	-19.35	10	3.06	13	4.40	13
Bank Variables	-26.64	7	-17.04	10	-8.98	9
Halkbank						
Unconditional	-20.81	13	-22.64	12	-7.29	10
Macroeconomic	-15.81	11	-9.27	5	-5.79	6
Bank Variables	-19.41	11	-14.24	11	-8.81	10
Vakifbank						
Unconditional	-42.89	3	-25.86	9	-16.31	7
Macroeconomic	-9.62	13	-5.92	9	-4.47	9
Bank Variables	-27.31	6	-21.72	6	-12.59	8
Isbank						
Unconditional	-25.43	8	-28.25	7	-21.82	3
Macroeconomic	-14.82	12	-9.59	4	-7.09	4
Bank Variables	-18.73	12	-18.69	9	-14.67	7
Akbank						
Unconditional	-25.03	10	-28.11	8	-20.37	4
Macroeconomic	-20.11	9	-13.24	1	-13.83	1
Bank Variables	-16.86	13	-19.86	8	-18.34	5
Garanti						
Unconditional	-24.06	12	-30.34	5	-17.77	6
Macroeconomic	-27.38	7	-6.72	8	-8.07	2
Bank Variables	-29.09	5	-35.16	3	-33.75	2
Yapi Kredi						
Unconditional	-32.69	6	-34.32	2	-26.28	1
Macroeconomic	-26.41	8	-11.42	2	-7.52	3
Bank Variables	-40.17	3	-41.09	1	-35.36	1
Denizbank						
Unconditional	-34.38	5	-28.41	6	-6.42	11
Macroeconomic	-28.53	6	-8.90	6	-4.57	7
Bank Variables	-22.76	10	-21.51	7	-5.14	11
Finansbank						
Unconditional	-24.73	11	-25.03	11	-4.58	13
Macroeconomic	-32.78	4	-7.67	7	-4.50	8
Bank Variables	-24.25	9	-12.61	12	4.63	13
TEB						
Unconditional	-59.85	1	-34.22	3	-21.92	2
Macroeconomic	-60.16	1	-10.20	3	-6.67	5
Bank Variables	-65.20	1	-37.96	2	-31.78	3
Sekerbank						
Unconditional	-49.71	2	-31.04	4	-18.85	5
Macroeconomic	-33.26	3	-2.45	11	2.26	11
Bank Variables	-38.51	4	-32.86	4	-22.95	4
TSKB						
Unconditional	-31.63	7	-34.57	1	-12.97	9
Macroeconomic	-35.89	2	-1.90	12	2.41	12
Bank Variables	-26.00	8	-28.3	5	-15.74	6
Kalkinma						
Unconditional	-36.19	4	-19.66	13	-4.91	12
Macroeconomic	-30.99	5	-4.49	10	-2.53	10
Bank Variables	-40.51	2	-10.90	13	3.63	12

Looking at the average VaR5% values, we can conclude that TEB has the highest VaR (as an absolute value of 59.85%) value, whereas Halkbank has the lowest. The VaR value calculated for Halkbank as 20.81% for the 5^th^ quantile indicates that the loss to be experienced in the asset returns of Halkbank for a given quarter will not be more than 20.81% as an absolute value with 95% probability. As explained above, CoVaR gives the maximum loss incurred by the financial system when a bank return is at its VaR5% level. Thus, high values of CoVaR show high spillover effects on the financial system. According to average CoVaR5% results, the largest spillover affects to the financial system seem to arise from TSKB, Yapi Kredi and TEB, while the lowest come from Kalkinma and Halkbank. The CoVaR value calculated for Halkbank indicates that if the return losses of Halkbank reach the level of VaR5%, the estimated VaR5% value of the system would be 22.64%.

We observe that the banks with the lowest individual VaRs are not necessarily the banks with the lowest CoVaR values, such as Akbank, Garanti and Yapi Kredi. The opposite is also true; that is, the highest VaR value does not imply the highest CoVaR value. For example, Vakifbank has one of the highest VaR5% values, but one of the lowest CoVaR5% values. Therefore, even though the individual risk of Vakifbank seems to be the highest, its spillover risk to the financial system does not appear to be the highest. This result implies a loose relation between VaR and CoVaR. These findings are consistent with previous researches, primarily the research by Adrian and Brunnermeier on "CoVaR" (2016), Lopez-Espinoza et al. (2012) and Lehar (2005).

ΔCoVaR shows how much the VaR of the financial system returns is affected when a bank's asset returns move from its median state to financial distress, i.e., from its VaR50% level to its VaR5% level. For example, ΔCoVaR for Halkbank was estimated as -7.29% for a quarter, which means that the VaR5% of the financial system would be increased by 7.29% (an absolute value) when the VaR value of Halkbank moves from its VaR50% level to financial distress, i.e., VaR5%. When we look at which banks have the greatest marginal contribution to the systemic risk of the financial system measured by ΔCoVaR, we find Yapi Kredi, TEB, Isbank and Akbank. The banks contributing the least are Finansbank and Kalkinma. This result is not surprising when take into consideration the size and scale of the international activity of Yapi Kredi, Isbank and Akbank since these banks are among the largest private banks in Turkey in terms of asset size.

ii According to the estimation results based on macroeconomic variables, we deduce the following:

TEB, TSKB and Sekerbank have the highest VaR values, whereas Vakifbank, Isbank and Halkbank have the lowest. The average VaR5% values for all the banks are larger than their average CoVaR5% values; that is, their spillover risk is lower than the banks’ individual risk. This result implies that the banks carry the highest individual risk when we take into consideration macrovariables. We observe that the largest contagion effects to the banking system, i.e., the largest CoVaR values, appear to come from Akbank, Yapi Kredi, TEB and Isbank, whereas the lowest arise from Ziraat and TSKB.

According to the ΔCoVaR results, Akbank, Garanti, Yapi Kredi and Isbank have the highest systemic risk contribution to the financial system. Ziraat would be considered the most stable bank. This result is expected since these four banks are among the largest private banks in Turkey with respect to asset size and variety of financial activities, so they are strongly correlated with the international financial market.

iii According to the estimation results based on individual bank variables, the following is concluded:

TEB again has the highest VaR value, as in the others. While Yapi Kredi has the highest CoVaR5% value, Kalkinma has the lowest. The average CoVaR5% estimation results for Akbank, Garanti, Yapi Kredi and TSKB are larger than their average VaR5% values. This finding implies that their spillover effects on the financial system are larger than their individual risks. This result may be explained by the fact that Akbank, Garanti and Yapi Kredi are more correlated with the financial system than Kalkinma. The estimation values of CoVaR and VaR for Isbank are relatively the same.

The empirical results show that Yapi Kredi, Garanti, TEB, Sekerbank and Akbank have the highest marginal systemic risk contributions to the financial system, while Kalkinma and Finansbank have the lowest. The average ΔCoVaR is higher than the average VaR only for the banks Akbank and Garanti. Moreover, their CoVaR values are higher than their individual risk. This finding implies that these two banks have greater contributions to the systemic risk of the financial system and that they cause the largest negative externalities. While Akbank has the lowest VaR5% value in terms of its bank variables, it has one of the highest values of both CoVaR and ΔCoVaR. The estimation results of Akbank provide a good example for presenting the loose relation between VaR and CoVaR as well as between VaR and ΔCoVaR.

In conclusion, Yapi Kredi, Garanti, Akbank, Isbank, TEB and Vakifbank seem to have the highest systemic risk rankings based on three sets of variables in terms of CoVaR and ΔCoVaR. When we review all the results, to identify the systemically important banks is a difficult decision since which model or which ranking can be used is not certain. From the regulators point of view, the ΔCoVaR rankings based on bank-specific variables come into prominence since BCBS states that the negative impact of domestic systemically important banks (D-SIBs) on the local economy should be assessed having regard to bank-specific factors which are size, interconnectedness, substitutability and complexity in the framework for dealing with D-SIBs (BCBS, 2012). Thus, the regulator authorities should follow the BCBS suggestion and use bank-specific variables to specify the systemically important banks of Turkey.

We find a few studies that examine the systemic risk contributions of Turkish banks on a bank level in the existing literature. The current studies generally investigate the determinants of systemic risk affecting to the Turkish financial system. We have a few alternatives in order to compare our results to the existing studies. Sacci and Sayılgan (2014) ranked twenty-eight Turkish banks in terms of their systemic risk importance using indicator-based method, and concluded that Isbank, Garanti, Akbank, Ziraat, Yapi Kredi, Vakifbank and Halkbank were identified as the systemically important banks of Turkey in descending order. In our study, we also find that Yapi Kredi, Akbank, Garanti, Isbank and TEB are identified as the most systemically important banks. Sengul and Yilmaz (2019) who ranked six Turkish banks based on their stock returns by using the CoVaR and MES methods. Finansbank, ICBC Turkey Bank, Yapi Kredi, Akbank, Garanti and Isbank were ranked according to their systemic risk contribution in descending order. Their list started with the smaller banks such as Finansbank in terms of asset size. However, our results put the four largest banks in Turkey at the top in our list.

## 6 Conclusions and discussion

The aim of this study is to measure systemic risk contributions of Turkish banks and to identify the banks contributing the most to the systemic risk of the financial system. The CoVaR method first proposed by Adrian and Brunnermeier (2009) was applied using quantile regression as a measurement method of systemic risk. The dataset used in the study includes quarterly asset returns of thirteen banks and quarterly state variables during the period from January 2005 to December 2016. The quarterly macroeconomic variables including BIST100 and volatility and bank variables consisting of tier 1 capital ratio, capital adequacy ratio, leverage and liquid assets ratios were added to the model as state variables. We tested the CoVaR method by adding state variables into the CoVaR model in addition to the return of a bank for capturing time variation. The bank with the maximum CoVaR is considered to increase the systemic risk of the financial sector and have negative spillover effects on the economy. We also evaluated the banks with the highest marginal contribution to the systemic risk of the Turkish banking system with respect to state variables. The systemic risk contribution of a financial institution to the system is denoted by ΔCoVaR; the higher ΔCoVaR is, the greater the contribution to the systemic risk. Moreover, we created a ranking list of banks corresponding to their VaR, CoVaR and ΔCoVaR estimation results.

The main findings from all the results are that even if the smallest banks in terms of asset size generally have the highest VaR values, they also have the smallest CoVaR and ΔCoVaR values. This finding implies that these banks carry substantial idiosyncratic risks, whereas their systemic risk contributions to the system are rather small, including those of Denizbank, Finansbank, Sekerbank, TSKB and Kalkinma.

With regard to the results of state variables, BIST100 seems to be a good predictor for estimating the systemically important banks as BIST100 has a positively significant relationship to the asset returns of all the banks. Furthermore, we examine the relation between capital measured by the ratios of Tier 1 capital and capital adequacy and systemic stability. The general thought is that capital shortage is associated with higher systemic risks. We find that *Tier 1* capital ratio is generally significant and has a greater impact on reducing spillover risk measured by CoVaR to the financial market. Our results show that this ratio exhibits a negative relationship with the stock returns in general; that is, low level of Tier 1 capital leads to higher return losses, but the reverse is true for capital adequacy ratio. This may be due to the components of Tier 1 capital since it has a higher quality capital according to the Basel rules. The asset returns of large banks such as Ziraat, Halkbank and Garanti appear to be more sensitive to the leverage ratio than small banks in terms of asset size, such as Sekerbank and Kalkinma. The coefficients of liquidity and leverage in the CoVaR model are generally negative which implies that higher levels of liquidity and leverage reduce systemic risk.

We observe that the CoVaR and ΔCoVaR values of the banks based on individual bank variables are larger than those of the values with respect to macroeconomic variables. This finding indicates that the banks’ balance-sheet characteristics have a greater impact on the contagion effect of the banks and their systemic risk contributions to the system as well. The individual bank variables appear to increase the vulnerabilities of the banks against to the crises rather than macroeconomic variables.

The empirical results reveal that Yapi Kredi, Garanti, Akbank, Isbank, TEB and Vakifbank have the greatest contribution to the to the systemic risk of the Turkish banking sector based on three sets of variables and thus appear to be the most systemically important banks in Turkey. Among those banks, Akbank, Garanti, Yapi Kredi and Isbank, the four largest private banks in Turkey in terms of asset size, have the highest systemic risk rankings as expected since they operate on a global scale and thus have strong relationships with the global financial market.

When reviewing the results of public banks, Ziraat, the largest bank in Turkey, seems to be the most stable bank. All the VaR values of Ziraat are larger than their CoVaR values. This finding implies that Ziraat has a larger individual risk than its spillover effects on the banking system. According to ΔCoVaR values, we observe that the systemic risk contribution of Ziraat is increased when taking into consideration its individual bank variables. It appears that although public banks have the smallest VaR values, they have relatively higher ΔCoVaR values, and Vakifbank seems to carry a more systemic risk contribution to the financial market than that carried by the other public banks.

We conclude that the individual risks and the spillover risk of public banks are lower than those of private banks. Only one exception is found, namely, the idiosyncratic risk of Vakifbank, which is one of the largest estimations. Again, we observe that the marginal systemic risk contributions of public banks are lower than those private banks. This finding indicates a lower influence of public banks on the instability of the Turkish banking sector during a crisis. These results conflict with the general consensus, since public banks of Turkey are expected to provide higher systemic risk contributions to the financial system. One of the possible explanations for this result is that public banks have cost and profit advantages over private banks in Turkey, as they have a valuable advantage in the ability to reserve public deposits of all government authorities and public companies without paying interest or paying less. This advantage would strengthen their capital structure and reduce the possibility of their failure in the event of a crisis and would benefit financial stability.

The results of the study can be used for potential policy implications. First, we find that capital is an important predictor for the systemic risk contribution of a bank. Due to the uncontrolled risk arising from the interconnected structure of the financial system, systemic risk leads to a substantial liquidity squeeze, liquidity deficiency and capital losses in a crisis situation both in the financial system and in financial institutions. Indeed, our results suggest that higher capital does eliminate the negative effects of systemic risk. Our results also show the importance of the type of capital. The authorities should give more emphasis to higher quality capital, i.e., Tier 1 capital. Second, the empirical results suggest that leverage ratio appears to be a substantial predictor for large banks. Third, although this study does not include bank size as a predictor to examine its effect to the systemic risk of the financial market, our results support the view that larger banks impose larger systemic risk contributions to the banking system. In conclusion, strict supervision of the capital requirements of banks, monitoring bank size and leverage ratio especially for large banks should be taken into consideration by the existing regulatory authorities in order to mitigate the adverse effects of systemic risk. For controlling the systemic risk of the Turkish banking sector, the authorities should also design the regulatory issues in accordance with the context of ΔCoVaR besides monitoring the idiosyncratic risks of banks.

## Declarations

### Author contribution statement

Z. Civan: Performed the experiments; Analyzed and interpreted the data; Contributed reagents, materials, analysis tools or data; Wrote the paper.

G. G. Simsek: Conceived and designed the experiments; Analyzed and interpreted the data; Contributed reagents, materials, analysis tools or data.

E.C. Akay: Conceived and designed the experiments.

### Funding statement

This research did not receive any specific grant from funding agencies in the public, commercial, or not-for-profit sectors.

### Competing interest statement

The authors declare no conflict of interest.

### Additional information

No additional information is available for this paper.

## Figures and Tables

**Table:** 

Banks	Abbrev.	Founding Year	2005 %	2006 %	2007 %	2008 %	2009 %	2010 %	2011 %	2012 %	2013 %	2014 %	2015 %	2016 %
T.C. Ziraat Bankası A.Ş.	Ziraat	1863	16.39	14.83	14.42	14.79	15.59	15.72	13.84	12.55	12.69	13.11	13.54	13.78
T. Halk Bankası A.Ş.	Halkbank	1938	6.81	7.10	7.17	7.24	7.60	7.58	7.85	8.34	8.56	8.23	8.40	8.92
T. Vakıflar Bankası T.A.O.	Vakifbank	1954	8.16	7.64	7.56	7.39	8.11	7.69	7.68	8.06	8.29	8.38	8.18	8.19
T. İş Bankası A.Ş.	Isbank	1924	16.05	15.51	14.29	13.82	14.18	13.70	13.93	13.51	12.87	12.59	12.33	12.01
Akbank T.A.Ş.	Akbank	1948	13.20	11.81	12.15	12.13	11.94	11.77	11.51	12.01	11.24	10.88	10.50	10.44
T. Garanti Bankası A.Ş.	Garanti	1946	9.19	10.37	12.04	12.60	13.21	12.89	12.63	12.34	12.04	11.59	11.37	10.95
Yapı Kredi Bankası A.Ş.	Yapi Kredi	1944	6.01	10.08	8.97	9.03	8.08	8.81	9.31	9.41	9.10	9.60	9.86	9.74
Denizbank A.Ş.	Denizbank	1997	2.36	2.37	2.66	2.72	2.66	2.88	3.10	3.40	3.63	3.68	3.77	3.97
Finans Bank A.Ş.	Finansbank	1987	3.10	3.69	3.72	3.76	3.67	3.96	3.98	4.19	4.04	3.98	3.83	3.91
Türk Ekonomi Bankası A.Ş.	TEB	1927	1.37	1.71	2.10	2.09	1.89	1.98	3.28	3.35	3.27	3.34	3.22	3.07
Şekerbank T.A.Ş.	Sekerbank	1953	0.79	0.83	1.08	1.14	1.12	1.18	1.24	1.12	1.15	1.12	1.09	0.92
T. Sınai Kalkınma Bankası A.Ş.	TSKB	1950	0.84	0.84	0.87	0.88	0.86	0.82	0.81	0.79	0.79	0.83	0.93	0.92
T. Kalkınma Bankası A.Ş.	Kalkinma	1975	0.17	0.18	0.15	0.15	0.16	0.17	0.24	0.22	0.22	0.21	0.21	0.27

Note: Ziraat, Halkbank and Vakifbank are public banks. Kalkinma is a state-owned development bank whose share is rather small. The rest of the banks are private banks.

Source: The Banks Association of Turkey (TBB).

**Table:** 

Variable	Symbol	Definiton	Source
Asset Return of a bank	Xi	The quarterly return of market valued total assets of a financial institution-i	TBB and the banks' websites
Return of BIST100	BIST100	BIST100 refers to a well-known indicator index of Borsa İstanbul A.Ş. denoted by XU100. The quarterly Return of BIST100 is calculated from the 2nd session closing BIST100 return index data.	Borsa Istanbul A.Ş.
Return of Equity Volatility	Volatiliy	The equity volatility is a value which is calculated as the standard deviation of the market over 63 days of BIST100 index based on 21 days in every month. The return of equity volatility represents the change in equity volatility. The euqity volatility data are not calculated. The equity volatility data are obtained from Borsa Istanbul A.Ş.	Borsa Istanbul A.Ş.
Tier1 Capital Ratio	Tier 1	Tier 1 capital ratios of the banks are obtained from the independent audit reports of the banks, and they are not recalculated.	TBB and the banks' websites
Capital Adequacy Ratio	CAR	Capital adequacy ratios of the banks are obtained from the independent audit reports of the banks, and they are not recalculated.	TBB and the banks' websites
Liquid Assets Ratio	Liquidity	This ratio was calculated for each bank using the banks' balance sheets as of the end of quarters by the following formula: Liquid Assets Ratio = Liquid Assets/Total Assets Liquid Assets = Cash and Balances with the Central Bank of Turkey + Financial assets where fair value change is reflected to income statement (net) + Banks + Money Market Placements + Financial Assets Available for Sale (Net)	The banks' balance sheets obtained from TBB and the banks' websites
Leverage Ratio	Leverage	Leverage ratio is calculated for each bank as of the end of quarter by the following formula: Leverage Ratio = Total Capital/Total Assets "Total Capital and Total Assets" in the banks' balance sheets as of the end-of-quarter were taken into consideration.	The banks' balance sheets obtained from TBB and the banks' websites
